# Low-temperature crystal structure of 4-chloro-1*H*-pyrazole

**DOI:** 10.1107/S2056989021008604

**Published:** 2021-08-24

**Authors:** Kelly Rue, Raphael G. Raptis

**Affiliations:** a Department of Chemistry and Biochemistry, 11200 SW 8th Street, Miami, FL 33199, USA

**Keywords:** crystal structure, pyrazole, proton disorder, low temperature

## Abstract

The structure of 4-chloro-1*H*-pyrazole, C_3_H_3_ClN_2_, at 170 K has ortho­rhom­bic (*Pnma*) symmetry and is isostructural to its bromo analogue. Data were collected at low temperature since 4-chloro-1*H*-pyrazole sublimes when subjected to the localized heat produced by X-rays. The structure displays inter­molecular N—H⋯N hydrogen bonding and the packing features a trimeric mol­ecular assembly bis­ected by a mirror plane (*m* normal to *b*) running through one chlorine atom, one carbon atom and one N—N bond. The asymmetric unit therefore consists of one and one-half 4-chloro-1*H*-pyrazole mol­ecules. Thus, the N—H proton is crystallographically disordered over two positions of 50% occupancy each.

## Abstract 2

The crystal structure of 4-chloro-1*H*-pyrazole has been determined at 170 K, showing a hydrogen-bonded trimeric mol­ecular assembly that is isostructural to its bromo analogue, 4-bromo-1*H*-pyrazole.

## Chemical context   

Pyrazoles are a family of five-membered, π-excess aromatic heterocycles with adjacent nitro­gen atoms (Krishnakumar *et al.*, 2011 ▸; Karrouchi *et al.*, 2018 ▸). They serve as scaffolds for numerous pharmaceutically active compounds and agrochemicals as they are stable and amenable to substitution (Naim *et al.*, 2016 ▸). They readily coordinate to metals, forming a large group of complexes where typically pyrazoles are monodentate or bridging bidentate pyrazolido ligands (Pettinari *et al.*, 2010 ▸; Viciano-Chumillas *et al.*, 2010 ▸). In the solid state, pyrazoles form hydrogen-bonded aggregates whose topology depends on the steric bulk of peripheral substituents and the acidity of the N1—H proton and basicity of the N2 atom. For example, 4-bromo-pyrazole and 3-methyl-pyrazole form approximately planar triangular assemblies (Foces-Foces *et al.*, 1999 ▸; Goddard *et al.*, 1999 ▸), 3,5-diphenyl-pyrazole and 3,5-bis­(tri­fluoro­meth­yl)pyrazole form tetra­nuclear assemblies (Raptis *et al.*, 1993 ▸; Alkorta *et al.*, 1999 ▸), while pyrazole forms a one-dimensional polymeric chain (Krebs Larsen *et al.*, 1970 ▸).
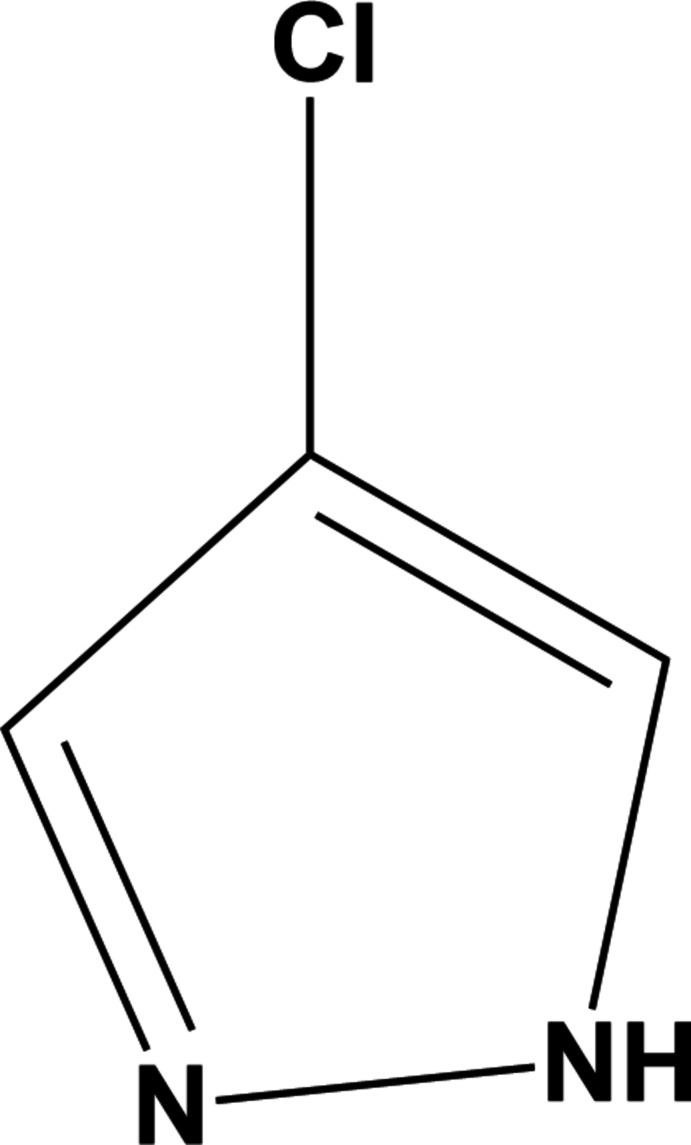


## Structural commentary   

**Figure 1:**
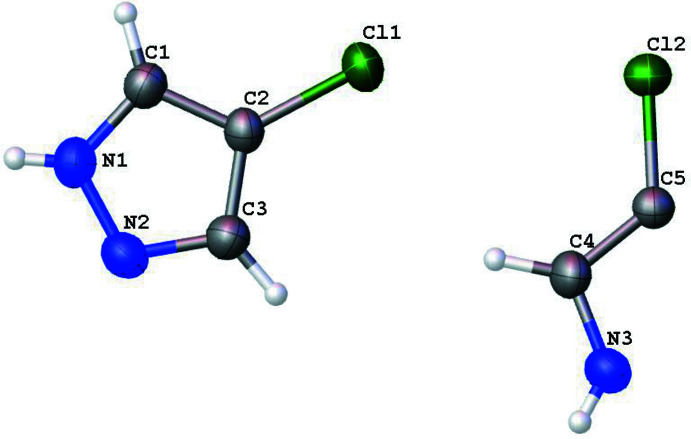
Perspective view of the asymmetric unit of 4-chloro-1*H*-pyrazole. Displacement ellipsoids are shown at 50% probability and half the disordered protons are removed for clarity.

**Figure 2:**
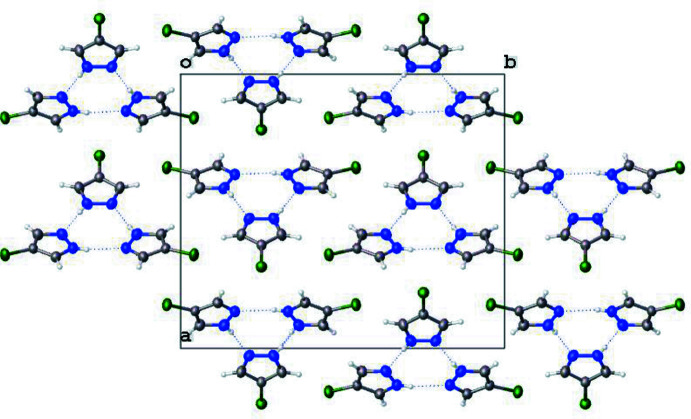
Packing of 4-chloro-1*H*-pyrazole, viewed parallel to the *c* axis, showing the formation of trimeric units. Half the disordered protons have been removed for clarity.

4-Chloro-1*H*-pyrazole crystallizes with one and one-half mol­ecules in the asymmetric unit (*Z*′ = 1.5) as shown in Fig. 1 ▸. The second mol­ecule is bis­ected by a mirror plane normal to *b* (*x*, 3/4, *z*), which runs through C5, Cl2, and the N3—N3^i^ bond [symmetry code: (i) *x*, 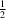 − *y*, *z*]. As a result of this mirror plane, the NH protons are crystallographically disordered over two positions. As with the bromo analogue (Foces-Foces *et al.*, 1999 ▸), 4-chloro-1*H*-pyrazole crystallizes in the *Pnma* space group and forms trimeric units (Fig. 2 ▸).

Also disordered in this structure are the C and N atoms. Without disorder, pyrazoles have one ‘pyrrole-like’ side and one ‘pyridine-like’ side, as was discussed in the neutron diffraction study of 1*H*-pyrazole (Krebs Larsen *et al.*, 1970 ▸). The C—N, C—C, and C—H bonds on either side of the mol­ecule are crystallographically distinct and resemble either pyrrole or pyridine. However, due to the disorder of the N—H protons in the current structure, only average positions of the C and N atoms have been obtained. Therefore, the C—N, C—C, and C—H bonds on either side of the mol­ecule are indistinguishable. This is most apparent in the C—N bonds. In the current structure, the C—N bonds are 1.335 (2), 1.334 (2), and 1.334 (2) Å for C1—N1, C3—N2, and C4 —N3, respectively. In the 1*H*-pyrazole structure, the C—N bond lengths are 1.356 and 1.350 Å for the ‘pyrrole-like’ side and the ‘pyridine-like’ side, respectively. The C—N bond lengths of the current structure are in agreement with those previously reported in the 4-bromo analogue with C—N bond lengths of 1.343 (10), 1.331 (10), and 1.327 (10) Å (Foces-Foces *et al.*, 1999 ▸). Furthermore, these bond lengths are also in agreement with the 4-chloro-1*H*-pyrazol-2-ium chloride salt, which exhibits C—N bond lengths of 1.334 (2) and 1.331 (2) Å (Farmiloe *et al.*, 2019 ▸). The N—N bonds of the present mol­ecule are 1.346 (2) and 1.345 (3) Å, for N1—N2 and N3—N3^i^, respectively, and are similar to those of 4-methyl-1*H*-pyrazole (which is refined without proton disorder in *Pca*2_1_) with N—N bond lengths of 1.343 (2), 1.344 (2), and 1.349 (2) Å (Goddard *et al.*, 1999 ▸). However, the N—N bonds of the 4-bromo analogue are slightly shorter at 1.335 (9) Å each (Foces-Foces *et al.*, 1999 ▸).

## Supra­molecular features   

**Figure 3:**
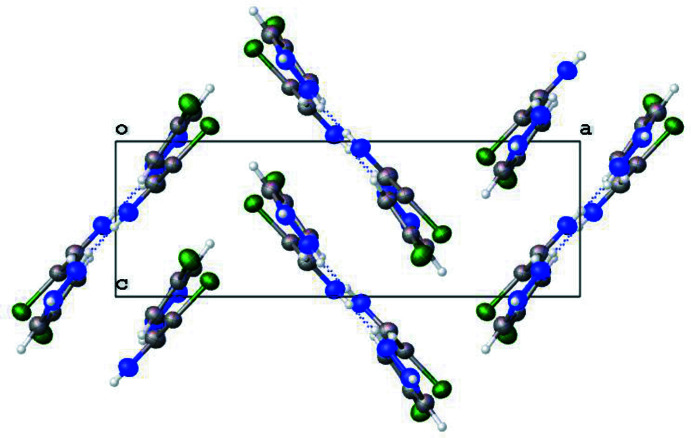
Packing of 4-chloro-1*H*-pyrazole, viewed parallel to the *b* axis, showing the formation of a herringbone motif.

Pyrazoles are known to crystallize in a variety of hydrogen-bonded motifs – dimers, trimers, tetra­mers, and catemers (Infantes & Motherwell, 2004 ▸; Pérez & Riera, 2009 ▸). The current structure of 4-chloro-1*H*-pyrazole crystallizes in trimeric units, as do the 4-bromo and 4-methyl analogues. The inter­molecular N1⋯N1^i^, N2⋯N3^ii^, and N3⋯N2^ii^ [symmetry codes: (i) *x*, −*y* + 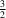, *z*; (ii) −*x* + 1, −*y* + 1, −*z*] are 2.885 (3), 2.858 (2), and 2.858 (2), respectively, as shown in Table 1 ▸. These values are in agreement with other inter­molecular hydrogen-bond inter­actions of pyrazoles. Just as with the 4-bromo derivative, 4-chloro-1*H*-pyrazole packs in a herringbone arrangement when viewed down the *b* axis (Fig. 3 ▸). The current structure exhibits no π-stacking inter­actions.

## Synthesis and crystallization   

4-Chloro-1*H*-pyrazole was purchased commercially and crystals were grown from the slow evaporation of a methyl­ene chloride solution.

## Refinement   

Crystal data, data collection and structure refinement details are summarized in Table 2 ▸. The data were collected at 170 K on a Bruker D8 CMOS diffractometer equipped with a Photon II detector. *CrysAlis PRO* was used for scaling and absorption correction. All C—H protons were refined freely while the N—H protons were fixed using an AFIX command and constrained to half occupancy due to the proton disorder.

To remove the proton disorder, an attempt was made to refine the mol­ecule in the non-centrosymmetric *Pn*2_1_ *a* space group, which is also consistent with the systematic absences. In *Pn*2_1_ *a*, hydrogen atoms were refined in both possible positions – on the odd-labeled N atoms or on the even-labeled N atoms. However, as Foces-Foces *et al.* (1999 ▸) found with the 4-bromo-analogue, refinement in the lower symmetry space group did not improve the refinement. For the trial *Pn*2_1_ *a* structure with protons on the even-labeled N atoms, the *R*_1_ and *wR*_2_ values slightly increased to 3.67% and 9.67%, respectively, and the shifting of the carbon atoms could not be consolidated. For the trial *Pn*2_1_ *a* structure with protons on the odd-labeled N atoms, the *R*_1_ and *wR*_2_ values increased even more to 3.71% and 9.69%, respectively, and the coordinates of the non-proton­ated N atoms could not converge. Therefore, the best refined model was chosen to be in the centrosymmetric *Pnma* space group with NH protons disordered over two positions.

## Supplementary Material

Crystal structure: contains datablock(s) I. DOI: 10.1107/S2056989021008604/zl5013sup1.cif

Structure factors: contains datablock(s) I. DOI: 10.1107/S2056989021008604/zl5013Isup2.hkl

Supporting information file. DOI: 10.1107/S2056989021008604/zl5013Isup3.cml

CCDC reference: 2103822

Additional supporting information:  crystallographic information; 3D view; checkCIF report

## Figures and Tables

**Table 1:** Hydrogen-bond geometry (Å, °)

*D*—H⋯*A*	*D*—H	H⋯*A*	*D*⋯*A*	*D*—H⋯*A*
N1—H1*A*⋯N1^i^	0.88	2.03	2.885 (3)	165
N2—H2⋯N3^ii^	0.88	1.99	2.8582 (19)	169
N3—H3*A*⋯N2^ii^	0.88	1.99	2.8582 (19)	169

Symmetry codes: (i) x, -y+{\script{3\over 2}}, z; (ii) -x+1, -y+1, -z.

**Table 2:** Experimental details

Crystal data	
Chemical formula	C_3_H_3_ClN_2_
*M*_r_	102.52
Crystal system, space group	Orthorhombic, *P* *n* *m* *a*
Temperature (K)	170
*a*, *b*, *c* (Å)	14.9122 (10), 17.6410 (9), 4.9878 (3)
*V* (Å^3^)	1312.13 (14)
*Z*	12
Radiation type	Mo *K*α
μ (mm^−1^)	0.69
Crystal size (mm)	0.29 × 0.14 × 0.08

Data collection	
Diffractometer	Bruker D8 CMOS
Absorption correction	Multi-scan (*CrysAlis PRO*; Rigaku OD, 2019 ▸)
*T*_min_, *T*_max_	0.760, 1.000
No. of measured, independent and observed [*I* > 2σ(*I*)] reflections	25891, 1798, 1481
*R*_int_	0.043
(sin θ/λ)_max_ (Å^−1^)	0.682

Refinement	
*R*[*F*^2^ > 2σ(*F* ^2^)], *wR*(*F* ^2^), *S*	0.033, 0.092, 1.06
No. of reflections	1798
No. of parameters	97
H-atom treatment	H atoms treated by a mixture of independent and constrained refinement
Δρ_max_, Δρ_min_ (e Å^−3^)	0.27, −0.26

Computer programs: *APEX3* (Brker, 2020 ▸), *CrysAlis PRO* (Rigaku OD, 2019 ▸), *SHELXS* (Sheldrick, 2008 ▸), *SHELXL2018*/3 (Sheldrick, 2015 ▸), and *OLEX2* (Dolomanov *et al.*, 2009 ▸).

## supplementary crystallographic information

### Crystal data

**Table:**

C_3_H_3_ClN_2_	*D*_x_ = 1.557 Mg m^−^^3^
*M**_r_* = 102.52	Mo *K*α radiation, λ = 0.71073 Å
Orthorhombic, *P**n**m**a*	Cell parameters from 6701 reflections
*a* = 14.9122 (10) Å	θ = 2.3–30.2°
*b* = 17.6410 (9) Å	µ = 0.69 mm^−^^1^
*c* = 4.9878 (3) Å	*T* = 170 K
*V* = 1312.13 (14) Å^3^	Rect. Prism, clear colourless
*Z* = 12	0.28 × 0.14 × 0.08 mm
*F*(000) = 624	

### Data collection

**Table:**

Bruker D8 CMOS diffractometer	1481 reflections with *I* > 2σ(*I*)
ω scans	*R*_int_ = 0.043
Absorption correction: multi-scan (CrysAlisPro; Rigaku OD, 2019)	θ_max_ = 29.0°, θ_min_ = 2.3°
*T*_min_ = 0.760, *T*_max_ = 1.000	*h* = −20→20
25891 measured reflections	*k* = −23→23
1798 independent reflections	*l* = −6→6

### Refinement

**Table:**

Refinement on *F*^2^	Primary atom site location: dual
Least-squares matrix: full	Secondary atom site location: difference Fourier map
*R*[*F*^2^ > 2σ(*F*^2^)] = 0.033	Hydrogen site location: mixed
*wR*(*F*^2^) = 0.092	H atoms treated by a mixture of independent and constrained refinement
*S* = 1.06	*w* = 1/[σ^2^(*F*_o_^2^) + (0.0418*P*)^2^ + 0.5547*P*] where *P* = (*F*_o_^2^ + 2*F*_c_^2^)/3
1798 reflections	(Δ/σ)_max_ = 0.001
97 parameters	Δρ_max_ = 0.27 e Å^−^^3^
0 restraints	Δρ_min_ = −0.26 e Å^−^^3^

### Special details

**Table:**

Geometry. All esds (except the esd in the dihedral angle between two l.s. planes) are estimated using the full covariance matrix. The cell esds are taken into account individually in the estimation of esds in distances, angles and torsion angles; correlations between esds in cell parameters are only used when they are defined by crystal symmetry. An approximate (isotropic) treatment of cell esds is used for estimating esds involving l.s. planes.

### Fractional atomic coordinates and isotropic or equivalent isotropic displacement parameters (Å^2^)

**Table:**

	*x*	*y*	*z*	*U*_iso_*/*U*_eq_	Occ. (<1)
Cl1	0.65912 (3)	0.45830 (2)	0.73672 (9)	0.04510 (15)	
C1	0.66638 (11)	0.61504 (9)	0.6893 (4)	0.0375 (3)	
C2	0.63595 (10)	0.54554 (8)	0.6008 (3)	0.0330 (3)	
N1	0.63427 (9)	0.66822 (7)	0.5248 (3)	0.0390 (3)	
H1A	0.644141	0.717242	0.538844	0.047*	0.5
C3	0.58468 (11)	0.56001 (10)	0.3776 (3)	0.0389 (4)	
N2	0.58435 (9)	0.63462 (8)	0.3340 (3)	0.0405 (3)	
H2	0.556236	0.657939	0.202649	0.049*	0.5
Cl2	0.70256 (4)	0.250000	0.59928 (11)	0.03892 (16)	
C5	0.62343 (15)	0.250000	0.3495 (4)	0.0326 (4)	
N3	0.52730 (9)	0.28813 (7)	0.0432 (3)	0.0395 (3)	
H3A	0.494432	0.317237	−0.061095	0.047*	0.5
C4	0.58543 (12)	0.31226 (10)	0.2276 (3)	0.0396 (4)	
H3	0.5571 (14)	0.5266 (11)	0.253 (4)	0.043 (5)*	
H1	0.7031 (12)	0.6279 (11)	0.839 (4)	0.040 (5)*	
H4	0.5962 (12)	0.3661 (12)	0.261 (4)	0.051 (6)*	

### Atomic displacement parameters (Å^2^)

**Table:**

	*U*^11^	*U*^22^	*U*^33^	*U*^12^	*U*^13^	*U*^23^
Cl1	0.0525 (3)	0.0303 (2)	0.0525 (3)	0.00392 (16)	−0.00952 (19)	0.00342 (16)
C1	0.0379 (8)	0.0335 (8)	0.0412 (8)	−0.0017 (6)	−0.0027 (7)	−0.0020 (7)
C2	0.0340 (7)	0.0281 (7)	0.0371 (8)	0.0020 (6)	0.0008 (6)	−0.0006 (6)
N1	0.0408 (7)	0.0330 (6)	0.0431 (7)	−0.0007 (6)	0.0017 (6)	−0.0012 (6)
C3	0.0429 (9)	0.0345 (8)	0.0391 (8)	0.0028 (6)	−0.0053 (7)	−0.0032 (7)
N2	0.0450 (8)	0.0383 (7)	0.0384 (7)	0.0053 (6)	−0.0022 (6)	0.0028 (6)
Cl2	0.0416 (3)	0.0395 (3)	0.0356 (3)	0.000	−0.0053 (2)	0.000
C5	0.0359 (11)	0.0311 (10)	0.0309 (10)	0.000	−0.0011 (9)	0.000
N3	0.0432 (7)	0.0370 (7)	0.0384 (7)	0.0068 (6)	−0.0036 (6)	−0.0001 (6)
C4	0.0483 (10)	0.0306 (8)	0.0401 (8)	0.0052 (7)	−0.0035 (7)	−0.0040 (7)

### Geometric parameters (Å, º)

**Table:**

Cl1—C2	1.7169 (15)	N2—H2	0.8800
C1—C2	1.380 (2)	Cl2—C5	1.716 (2)
C1—N1	1.335 (2)	C5—C4	1.377 (2)
C1—H1	0.952 (19)	C5—C4^i^	1.377 (2)
C2—C3	1.374 (2)	N3—N3^i^	1.345 (3)
N1—H1A	0.8800	N3—H3A	0.8800
N1—N2	1.3457 (19)	N3—C4	1.334 (2)
C3—N2	1.334 (2)	C4—H4	0.98 (2)
C3—H3	0.951 (19)		
			
C2—C1—H1	130.6 (11)	N1—N2—H2	125.7
N1—C1—C2	108.04 (14)	C3—N2—N1	108.50 (14)
N1—C1—H1	121.4 (11)	C3—N2—H2	125.7
C1—C2—Cl1	127.15 (13)	C4^i^—C5—Cl2	127.11 (10)
C3—C2—Cl1	126.74 (12)	C4—C5—Cl2	127.11 (10)
C3—C2—C1	106.09 (14)	C4—C5—C4^i^	105.8 (2)
C1—N1—H1A	125.6	N3^i^—N3—H3A	125.7
C1—N1—N2	108.87 (13)	C4—N3—N3^i^	108.61 (9)
N2—N1—H1A	125.6	C4—N3—H3A	125.7
C2—C3—H3	131.0 (12)	C5—C4—H4	129.2 (11)
N2—C3—C2	108.49 (15)	N3—C4—C5	108.50 (15)
N2—C3—H3	120.2 (12)	N3—C4—H4	122.3 (11)
			
Cl1—C2—C3—N2	−178.73 (13)	N1—C1—C2—Cl1	178.76 (12)
C1—C2—C3—N2	0.03 (19)	N1—C1—C2—C3	0.01 (18)
C1—N1—N2—C3	0.07 (18)	Cl2—C5—C4—N3	179.74 (15)
C2—C1—N1—N2	−0.05 (18)	N3^i^—N3—C4—C5	−0.20 (16)
C2—C3—N2—N1	−0.06 (19)	C4^i^—C5—C4—N3	0.3 (3)

Symmetry code: (i) *x*, −*y*+1/2, *z*.

### Hydrogen-bond geometry (Å, º)

**Table:**

*D*—H···*A*	*D*—H	H···*A*	*D*···*A*	*D*—H···*A*
N1—H1*A*···N1^ii^	0.88	2.03	2.885 (3)	165
N2—H2···N3^iii^	0.88	1.99	2.8582 (19)	169
N3—H3*A*···N2^iii^	0.88	1.99	2.8582 (19)	169

Symmetry codes: (ii) *x*, −*y*+3/2, *z*; (iii) −*x*+1, −*y*+1, −*z*.
